# Converting to Intubation During Non-intubated Thoracic Surgery: Incidence, Indication, Technique, and Prevention

**DOI:** 10.3389/fsurg.2021.769850

**Published:** 2021-10-26

**Authors:** Xu-Heng Chiang, Mong-Wei Lin

**Affiliations:** ^1^Department of Medical Education, National Taiwan University Hospital and National Taiwan University College of Medicine, Taipei, Taiwan; ^2^Department of Surgery, National Taiwan University Hospital and National Taiwan University College of Medicine, Taipei, Taiwan

**Keywords:** thoracic surgery, intubation, adverse effects, conversion, non-intubated surgery

## Abstract

Traditionally, intubated general anesthesia with one-lung ventilation is standard in thoracoscopic surgery. However, in recent decades, non-intubated thoracoscopic surgery (NITS) has become an alternative method to minimize the adverse effects of intubated general anesthesia. Non-intubated procedures result in fewer adverse events than tracheal intubation and general anesthesia, such as intubation-related airway injury, ventilation-induced lung injury, prolonged hospital stay, and postoperative nausea and vomiting. Despite these benefits, surgeons must consider the possibility of converting to intubation during NITS as the conversion rate is between 2 and 11%, varying between regions and learning time. The conversion rate is also affected by race, body size, the learning curve, and the surgical team's preferred methods. There are surgical (e.g., significant respiratory movements, uncontrolled bleeding, hindered surgical fields, large tumor sizes, adhesions) and anesthetic (e.g., hypoxemia, hypercapnia, airway spasms) reasons for converting to intubation. When a conversion is deemed necessary by the surgical team, the members should be well-prepared and act rapidly. Anesthesiologists should also feel comfortable intubating patients in the lateral decubitus position with or without bronchoscopic guidance. Patient selection is the key factor for avoiding conversion into an intubated surgery. Patients with an American Society of Anesthesiologists grade 2 or less, a body mass index <25, and less surgical complexity may be good candidates for NITS. Careful monitoring, adequate anesthesia depth, an experienced surgical team, and sufficient preparation can also prevent conversion. Conversion from a non-intubated into intubated thoracic surgery is unwanted but not inevitable. Therefore, NITS can be successful when performed on select patients by a well-prepared and experienced surgical team and is worthy of recommendation owing to its non-invasiveness.

## Introduction

Over the past few decades, there have been considerable advances in minimally invasive thoracoscopic surgery, including surgical techniques and anesthesia methods. Traditionally, deep general anesthesia and multi-portal video-assisted thoracic surgery (VATS) were used to minimize respiratory movement and reduce technical difficulty. As surgical skills improved, surgeons could tolerate diaphragmatic or mediastinal movement to some extent. As such, deep anesthesia is no longer necessary for VATS or uni-portal VATS, resulting in non-intubated thoracoscopic surgery (NITS) with spontaneous one-lung ventilation.

NITS is advantageous because it avoids perioperative morbidity derived from a mechanical ventilator and the unfavorable effects of deep anesthesia. For example, non-intubated anesthesia prevents potential damage caused by lung overdistension, the shear stress of repetitive opening and closing of alveoli, and the release of several inflammatory mediators (1–5). Subclinical lung injury caused by positive pressure ventilation also cannot be ignored, and minor respiratory impairment is associated with postoperative complications (6). NITS also prevents potentially fatal laryngeal or tracheal injuries from intubation (7, 8). The non-intubated method with adequate sedation can also reduce the use of analgesics, which can cause postoperative complications, including dizziness, vomiting, nausea, and respiratory depression (9). A residual neuromuscular block is another complication associated with muscle relaxants, eliciting postoperative acute airway events (e.g., hypoxemia, airway obstruction), unpleasant symptoms of muscle weakness, and delayed extubation (10, 11). The easy collapse of the dependent lung is another issue with mechanical ventilation and muscle paralysis, potentially increasing the risk of hypoxemia during one-lung ventilation. Furthermore, these deleterious effects may be more significant for susceptible patients (12).

The effectiveness of NITS is comparable with conventional intubated VATS, despite its non-invasiveness. Most comparative studies reported similar operative times and blood loss volumes (13–15). Currently, non-intubated methods have few technically unfavorable effects on surgeons, and the overall surgical time is shortened by not requiring anesthesia induction (16, 17).

Although non-intubated thoracic surgery has considerable benefits, there are some risks. First, hypercapnia and hypoxemia were frequently noted during NITS due to ineffective spontaneous one-lung ventilation (13), mostly in patients susceptible to systemic sedatives or with underlying lung diseases. Furthermore, anesthesiologists' control over the sedation depth is difficult because the patient's respiratory pattern must be suitable for the operation field and oxygenation maintenance (14). Barking, respiratory movements, or an elevated diaphragm may interfere with the surgical field if the depth of anesthesia is not under stationary control. When NITS started to become accepted in the early 2010s, some surgical teams used epidural anesthesia combined with intravenous narcotics (13, 18, 19). However, epidural anesthesia may be associated with a sympathetic blockade, perhaps leading to increased bronchial tone and airway hyper-reactivity (20). If these deleterious effects worsened during the operation, NITS was highly likely to be converted to an intubated surgery.

Conversion from NITS into an intubated surgery is unwanted. However, the correct timing and technical proficiency are crucial. Establishing appropriate selection criteria for non-intubated surgery and clear indications for conversion can better prepare the surgical team facing an unanticipated condition (Table 1) (27). Therefore, anesthesiologists and surgeons should be well-prepared and alert for possible conversions to ensure patient safety and the acceptability of NITS.

**Table 1:** Summary of non-intubated thoracoscopic surgery studies^a^.

**Studies**	**Country**	**Patient number**	**Mean age**	**Female (%)**	**Mean BMI**	**Conversion rate (L; S; W)**	**Reasons for conversion**	**Conversion risk factors**
Furak et al. (21)	Hungary	160	63.9	62.6	n/a	3.7% (L: n/a; S: n/a; W: n/a)	•Severe adhesions (50%) •Blood in airways (33.3%) •Serious diaphragm and mediastinal movements (16.7%)	n/a
Li et al. (22)	China	57	42.3	47.4	n/a	1.8% (L: n/a; S: n/a; W: n/a)	•Bleeding (100%)	n/a
Hung et al. (23)	Taiwan	1,025	59.3	73.0	22.6	2% (L: 4.7%; S: 2.7%; W: 0.3%)	•Considerable mediastinal movement (40%) •Bleeding (20%) •Poor epidural anesthesia (15%) •Persistent hypoxemia (15%) •Severe pleural adhesion (10%)	•BMI > 25 •Anatomical resection •Learning curve
Moon et al. (24)^b^	Korea	115	61.8	54.8	23.8	7.8% (L: n/a; S: n/a; W: n/a)	•Increased respiratory movement (77.8%) •Hypoxemia (33.3%) •Adhesion (22.2%) •Bronchospasm (11.1%)	•BMI •Age •Adhesion
Liu et al. (25)	China	339	55.4	50.3	22.5	5.6% (L: 7%; S: 0%; W: n/a)	•High degree of breathing and mediastinal flutter (28.6%) •Tumor invasion (28.6%) •Hypercapnia and hypoxemia (14.3%) •Pleural adhesions (14.3%) •Diaphragmatic lifting (14.3%) •Change of surgical method (14.3%) •Bleeding (14.3%)	n/a
Al-Abdullatief et al. (26)	Saudi Arabia	79	37.0	41.0	n/a	11% (L: n/a; S: n/a; W: n/a)	•Bleeding (22.2%) •Cardiac arrest (11.1%) •Hypercapnia (11.1%) •Diaphragm movement (11.1%) •Large tumor size (11.1%) •Adhesion (11.1%) •Conversion to pneumonectomy (11.1%)	n/a

a *We selected the study with largest patient cohort from several studies performed by the same group*.

b *This study included patients with pulmonary and mediastinal malignant or benign diseases*.

*L, lobectomy; S, segmentectomy; W, wedge; BMI, body mass index; n/a, not available*.

## Non-Intubated to Intubated Conversion Rate

The conversion rate from NITS into an intubated surgery is between 2 and 11% (21–26), differing based on centers and countries but primarily by learning time. NITS experience may be a key point affecting the conversion rate. Hung et al. reported a remarkable decrease in the conversion rate as the cumulative non-intubated case number increased over time (23). An ~10% conversion rate was reported by this surgical team in 2011 when they had performed 100–150 non-intubated surgeries (13). However, in 2018, when the team had >1,000 cases, the conversion rate improved to <3% (28, 29).

Other conversion risk factors were also reported, including older age, higher body mass index (BMI), and anatomical resection and adhesion (23, 24). However, there was no strict cutoff point for age as an NITS exclusion criterion.

Regarding BMI, researchers indicated patients with a BMI >25 or 30 are not ideal candidates for non-intubated anesthesia because more vigorous respiratory movement is expected, resulting in a disturbed surgical field (23, 24). The odds ratio for conversion into intubated VATS was reported up to 9.09 (range, 3.59–25.46) for patients with a BMI >25 (23). The conversion rates in different countries may come from variable baseline characteristics of the study population, including BMI, sex, and body size. For western studies including 50% or fewer female participants, a 5–10% conversion rate is relatively higher (26, 30) than the 2–5% reported by most studies from eastern countries (14, 18, 28, 29, 31, 32). Both ethnicity and sex were thought to be associated with body size and respiratory movement, which are often positively correlated in our experience.

Anatomical resection is another factor affecting the conversion rate. A previously published series reported a 0.3% conversion rate for wedge resection but 2.7 and 4.7% for segmentectomy and lobectomy, respectively (23). Chen et al. reported similar results in another series, in which the conversion rate was 1.3%, 7.1%, and 5.8% for wedge, segmentectomy, and lobectomy, respectively (31). The reported conversion rates were comparatively higher (5.6–10%) for studies that only enrolled patients undergoing an anatomical resection (13, 25).

Chronic lung distress could also impact conversion rate and compel surgeons not to perform NIST owing to higher risks and inefficiency. For example, an emphysematous lung would collapse more poorly and slowly in NITS procedures than intubated ones. Furthermore, NITS could not support respiratory function or intubation, and emergent conversion probably occurs. Although patients with impaired pulmonary function or chronic lung diseases are more susceptible to hypoxemia or hypercapnia, current literature reported no significant difference in conversion rate compared with general population (33). Wu et al. reported that NITS for lobectomy is technically feasible and as safe as thoracoscopic lobectomy performed with tracheal intubation in geriatric (≥65 years) patients with lung cancer (33). Even for patients with impaired pulmonary function (FEV15 = 7.9 ± 13.1%, FVC = 76.7 ± 13.8%), an intubation conversion rate of only 4% was reported. This conversion rate is similar to the results of other series. A series that enrolled >1,000 patients undergoing NITS also reported zero postoperative 30-day mortality rate among 20 patients who underwent intraoperative conversion to tracheal intubation (23). Therefore, NITS may be still applied as a safe alternative to intubated general anesthesia in highly selected patients.

There is no universal conversion rate in the literature because it is affected by numerous factors, such as the patient's baseline characteristics, surgical team's experience level, and complexity of the surgery.

## Conversion Indications

Under certain conditions, NITS may be converted into an intubated surgery to ensure patient safety and facilitate the surgical process. For surgeons, considerable mediastinal movement is the most common reason (~33.3–100%) to convert into intubated general anesthesia (15, 18, 24, 27, 28, 32, 34).

Strong mediastinal or respiratory movement can lead to hilum dissection difficulties and accidental injury to vital regions. Males with larger body sizes and heavier body weight are more likely to convert due to major respiratory fluctuation. Obesity is often associated with a higher respiratory rate and lower tidal volume and may directly cause large respiratory movements (35, 36).

Bleeding is another major reason for converting, accounting for ~12.5–33% of all intubated conversions (13, 18, 25, 26, 28, 31, 37). During NITS, accidental bleeding was often related to significant and unexpected respiratory movements, under-regulated cough reflexes, and hindered surgical fields caused by a non-collapsed lung. Conversion to intubated anesthesia makes respiratory movements controllable and reduces the surgeon's stress while protecting the contralateral airway if the bronchus was damaged.

For anesthesiologists, prolonged hypoxemia or hypercapnia generally elicits conversion from non-intubation into intubation. The cutoff criterion of hypoxemia varies among surgical groups. Some researchers have suggested converting if the oxygen saturation on pulse oximetry was <85% for more than 5 min (33). Others suggested that an oxygen partial pressure <60 mm Hg or carbon dioxide level >80 mm Hg should indicate conversion (38). The proportion of conversions resulting from hypoxemia or hypercapnia was between 14.3 and 33.3% (13, 24, 25, 37).

Disease characteristics, such as large tumor size or severe adhesion, have an important effect on the decision to convert; non-intubated anesthesia increases the surgical difficulty in already challenging cases. Further, the operative time may be longer for complicated cases, and NITS likely aggravates hypoxemia or hypercapnia. Disease-related conversions account for 14.3–50% (18, 21, 27). Other interfering factors adversely affecting non-intubated VATS include a non-collapsed lung, susceptibility to pain, airway hypersensitivity, and ineffective epidural anesthesia or intravenous sedation (13, 31, 37, 39).

The criteria of conversion from NITS to intubated VATS varies among different groups, according to their experience levels, maturity, and surgical techniques. Nevertheless, the general principles and situations of conversion are similar. The aforementioned indications of conversion can be summed up as surgical and anesthesiological aspects. Both these aspects have to be fully discussed among doctors when the situation gets severe, and the decision of conversion should be made jointly.

## Nits Conversion Technique

Despite strict patient selection criteria, converting to intubation is sometimes necessary due to unexpected events, such as hypoxemia, strong mediastinal movement, massive bleeding, or a hampered surgical field. Conversions are usually unpredictable and occur in an emergency. Therefore, surgeons and anesthesiologists should be well-prepared and establish appropriate conversion criteria.

Although it is technically demanding to intubate a patient in the decubitus position, it should not be unconquerable for an experienced anesthesiologist. In these situations, bronchoscope-guided intubation may help, and good patient selection should also minimize struggling during difficult intubations (32, 40). The Mallampati score is a good bedside indicator for potential obstacles in emergency intubation. A Mallampati score of 1 and an acceptable neck extension with a thyromental space extending more than four finger breadths indicate easier intubation, even in the decubitus position (41).

Hypoxemia and hypercapnia during spontaneous one-lung ventilation were common reasons for conversion. For these patients, the collapsed lung can be quickly re-expanded when the surgical wounds were sealed with transparent waterproof dressings after chest tube insertion (13). Double- and single-lumen endotracheal tubes with a blocker can be considered if conversion is deemed necessary. A laryngeal mask is also an option for the conversion, depending on the dexterity of the anesthesiologist (21). Some surgical teams rotate the table to insert endotracheal tubes in a relatively supine position.

We preferred straightforward intubation in the lateral decubitus position. Muscle relaxants (cisatracurium or rocuronium) were administered after sedatives such as propofol and fentanyl (30). Intubation was better assisted with video laryngoscope to facilitate the process. In our experiences, single-lumen intubation with blocker may be more efficient, but double-lumen intubation was also feasible.

Regardless, the anesthesiologist must be familiar with inserting an endotracheal tube, laryngeal mask, video-assisted intubation, and endobronchial blocker to choose the most suitable device depending on the patient's airway and position, the completion time of the procedure, and the causes for conversion. Together with well-established criteria and protocols, experienced team members are important for shortening the time from decision to intubation.

## Avoiding Conversion During Nits

Emergency intubation during conversion is usually undesirable, potentially resulting in airway injuries, prolonged operative time, and exacerbated stress of the team members. Therefore, avoiding conversion during NITS is ideal (8, 42). Table 2 summarizes the points to consider to avoid conversion during NITS.

**Table 2:** Considerations for avoiding conversions during non-intubated thoracoscopic surgery.

1. Patient selection Lower BMI (<25)^a^ ASA grade ( ≤ 2) Easier airway management (i.e., Mallampati score class 1, acceptable neck extension, etc.) No cardiopulmonary comorbidity
2. Less surgical complexity Early cancer stage Less adhesion expected No neoadjuvant therapies for cancerss
3. Anesthesia depth monitoring (BIS monitor)
4. Assistance with regional anesthesia Local wound infiltration Intercostal nerve blockade Vagal nerve blockade Thoracic paravertebral blocks and epidural blockade
5. Experienced surgical team Skilled surgeon Competent anesthesiologist Long run-in members

a *Some surgical groups reported a BMI <30 as a selection criterion. BMI, body mass index; ASA, American Society of Anesthesiologists; BIS, bispectral index*.

A preoperative patient briefing regarding the risks and benefits of the non-intubated method is necessary. Further, preoperative patient selection is crucial, and the attending surgeons should be exceptionally cautious and strict. Good candidates for NITS generally include those with a lower BMI, American Society of Anesthesiologists grades 1 or 2, and no cardiopulmonary issues (43, 44). The complexity of the surgical procedure also affects the feasibility of NITS. Although there is growing evidence that non-intubated anesthesia is safe and feasible for various thoracic procedures, from simple lung resections to complicated anatomical malignancy resections, it has generally been more acceptable to perform relatively simple procedures with the non-intubated technique (45–47). As the surgical skills and anesthesia techniques improve, non-intubated surgery will be applied more widely for moderately risky patients and difficult surgeries (26, 48–51).

Regarding the monitoring of non-intubated patients, a three-lead electrocardiogram, blood pressure monitoring, and pulse oximetry are minimally required. Monitoring airway patency, respiratory rate, and respiratory pattern during one-lung ventilation are also important. Careful monitoring can detect early signs that conversion is necessary and avoid emergency conversions.

The anesthesia depth should be carefully monitored and well-controlled, for example, by bispectral index monitoring, which helps guide anesthesiologists. Various levels of anesthesia depth were reported during non-intubated surgery. However, it is most important for the anesthesiologist to balance respiratory function and mediastinal movements (45, 47, 52–54). If a patient is over-sedated at the surgeon's request for less respiratory movement, hypoxemia and hypercapnia were likely to occur, leading to conversion and vice versa. Thus, adequate non-intubated surgery is based on a well-regulated depth of anesthesia with reasonable respiratory support, such as an oxygen mask or nasal high flow cannula (29).

Regional anesthesia techniques may also prevent conversions, including local wound infiltration, intercostal nerve blocks, vagal nerve blocks, thoracic paravertebral blocks (mostly erector spine plane), and epidural blocks. Regional blockades reduce the use of systemic sedatives and minimize respiratory sedation. Surgeons benefit from regional anesthesia because the cough reflex can be inhibited and fewer pain-stimulating movements (27). For NITS, sedation with regional analgesia is essential, indicating that a multi-pronged approach to analgesia is recommended.

Based on our experience, we shifted our regional anesthesia technique from epidural blocks to intercostal nerve blocks via direct thoracoscopic view. Epidural anesthesia may have risks of spinal cord injury, respiratory suppression, and hypotension (23). Intravenous anesthesia with regional anesthetic methods via direct thoracoscopic view, such as vagal block, intercostal block, and wound infiltration, is generally sufficient for the NITS procedure.

An inexperienced team is the absolute contraindication of non-intubated surgery (21). All team members should be accomplished and proficient in surgical and anesthesiological methodologies and informed of the potential complications and subsequent protocols. If a conversion is required, elective conversion is always preferable to emergency conversion; thus, all possible and subtle difficulties of every patient must be accessed and anticipated as early as possible. The equipment and drugs, including single- and double-lumen tubes, a bronchoscope, an endotracheal blocker, other advanced airway equipment, and first-aid medicine, must be prepared to avoid conversion failure.

## Conclusion

Conversion into intubated VATS is sometimes required during NITS. With correct patient selection, sufficient preparation, carefully established protocols, and skilled and rapidly acting surgical team members, the risks and complications of converting to intubation can be minimized to an acceptable level. NITS could be the treatment of choice for thoracic malignancies if physicians are proficient in preventing and managing potential conversions.

## Author Contributions

X-HC prepared the initial draft of this article, which was refined by M-WL. All authors contributed to the article and approved the submitted version.

## Funding

This work was supported by research grants from the National Taiwan University Hospital Taipei, Taiwan (NTUH110-S5037) and the Ministry of Science and Technology, Taiwan (MOST110-2314-B-002-271).

## Conflict of Interest

The authors declare that the research was conducted in the absence of any commercial or financial relationships that could be construed as a potential conflict of interest.

## Publisher's Note

All claims expressed in this article are solely those of the authors and do not necessarily represent those of their affiliated organizations, or those of the publisher, the editors and the reviewers. Any product that may be evaluated in this article, or claim that may be made by its manufacturer, is not guaranteed or endorsed by the publisher.

## References

[1] Della Rocca G Coccia C. Acute lung injury in thoracic surgery. Curr Opin Anaesthesiol. (2013) 26:40–6. 10.1097/ACO.0b013e32835c4ea2 23235524

[2] Schilling T Kozian A Huth C Buhling F Kretzschmar M Welte T. The pulmonary immune effects of mechanical ventilation in patients undergoing thoracic surgery. Anesth Analg. (2005) 101:957–65. 10.1213/01.ane.0000172112.02902.77 16192502

[3] Belperio JA Keane MP Lynch JP 3rd Strieter RM. The role of cytokines during the pathogenesis of ventilator-associated and ventilator-induced lung injury. Semin Respir Crit Care Med. (2006) 27:350–64. 10.1055/s-2006-948289 16909369

[4] Pavone LA Albert S Carney D Gatto LA Halter JM Nieman GF. Injurious mechanical ventilation in the normal lung causes a progressive pathologic change in dynamic alveolar mechanics. Crit Care. (2007) 11:R64. 10.1186/cc5940 17565688 PMC2206429

[5] Zupancich E Paparella D Turani F Munch C Rossi A Massaccesi S. Mechanical ventilation affects inflammatory mediators in patients undergoing cardiopulmonary bypass for cardiac surgery: a randomized clinical trial. J Thorac Cardiovasc Surg. (2005) 130:378–83. 10.1016/j.jtcvs.2004.11.061 16077402

[6] Gothard J. Lung injury after thoracic surgery and one-lung ventilation. Curr Opin Anaesthesiol. (2006) 19:5–10. 10.1097/01.aco.0000192783.40021.c1 16547427

[7] Fitzmaurice BG Brodsky JB. Airway rupture from double-lumen tubes. J Cardiothorac Vasc Anesth. (1999) 13:322–9. 10.1016/S1053-0770(99)90273-2 10392687

[8] Liu S Mao Y Qiu P Faridovich KA Dong Y. Airway Rupture Caused by Double-Lumen Tubes: A Review of 187 Cases. Anesth Analg. (2020) 131:1485–90. 10.1213/ANE.0000000000004669 33079871

[9] Hausman MS Jr Jewell ES Engoren M. Regional versus general anesthesia in surgical patients with chronic obstructive pulmonary disease: does avoiding general anesthesia reduce the risk of postoperative complications? Anesth Analg. (2015) 120:1405–12. 10.1213/ANE.0000000000000574 25526396

[10] Murphy GS Brull SJ. Residual neuromuscular block: lessons unlearned. Part I: definitions, incidence, and adverse physiologic effects of residual neuromuscular block. Anesth Analg. (2010) 111:120–8. 10.1213/ANE.0b013e3181da832d 20442260

[11] Murphy GS Szokol JW Avram MJ Greenberg SB Shear T Vender JS. Postoperative residual neuromuscular blockade is associated with impaired clinical recovery. Anesth Analg. (2013) 117:133–41. 10.1213/ANE.0b013e3182742e75 23337416

[12] Gruber EM Tschernko EM. Anaesthesia and postoperative analgesia in older patients with chronic obstructive pulmonary disease: special considerations. Drugs Aging. (2003) 20:347–60. 10.2165/00002512-200320050-00004 12696995

[13] Chen JS Cheng YJ Hung MH Tseng YD Chen KC Lee YC. Nonintubated thoracoscopic lobectomy for lung cancer. Ann Surg. (2011) 254:1038–43. 10.1097/SLA.0b013e31822ed19b 21869676

[14] AlGhamdi ZM Lynhiavu L Moon YK Moon MH Ahn S Kim Y. Comparison of non-intubated versus intubated video-assisted thoracoscopic lobectomy for lung cancer. J Thorac Dis. (2018) 10:4236–43. 10.21037/jtd.2018.06.163 30174869 PMC6105965

[15] Guo Z Yin W Pan H Zhang X Xu X Shao W. Video-assisted thoracoscopic surgery segmentectomy by non-intubated or intubated anesthesia: a comparative analysis of short-term outcome. J Thorac Dis. (2016) 8:359–68. 10.21037/jtd.2016.02.50 27076930 PMC4805789

[16] Hwang J Shin JS Son JH Min TJ. Non-intubated thoracoscopic bullectomy under sedation is safe and comfortable in the perioperative period. J Thorac Dis. (2018) 10:1703–10. 10.21037/jtd.2018.02.10 29707324 PMC5906215

[17] Zhang XX Song CT Gao Z Zhou B Wang HB Gong Q. A comparison of non-intubated video-assisted thoracic surgery with spontaneous ventilation and intubated video-assisted thoracic surgery: a meta-analysis based on 14 randomized controlled trials. J Thorac Dis. (2021) 13:1624–40. 10.21037/jtd-20-3039 33841954 PMC8024812

[18] Chen KC Cheng YJ Hung MH Tseng YD Chen JS. Nonintubated thoracoscopic lung resection: a 3-year experience with 285 cases in a single institution. J Thorac Dis. (2012) 4:347–51. 22934136 10.3978/j.issn.2072-1439.2012.08.07 PMC3426741

[19] Tseng YD Cheng YJ Hung MH Chen KC Chen JS. Nonintubated needlescopic video-assisted thoracic surgery for management of peripheral lung nodules. Ann Thorac Surg. (2012) 93:1049–54. 10.1016/j.athoracsur.2012.01.062 22386086

[20] Pace MM Sharma B Anderson-Dam J Fleischmann K Warren L Stefanovich P. Ultrasound-guided thoracic paravertebral blockade: a retrospective study of the incidence of complications. Anesth Analg. (2016) 122:1186–91. 10.1213/ANE.0000000000001117 26756911

[21] Furak J Szabo Z Tanczos T Paszt A Rieth A Nemeth T. Conversion method to manage surgical difficulties in non-intubated uniportal video-assisted thoracic surgery for major lung resection: simple thoracotomy without intubation. J Thorac Dis. (2020) 12:2061–9. 10.21037/jtd-19-3830 32642108 PMC7330381

[22] Li H Huang D Qiao K Wang Z Xu S. Feasibility of non-intubated anesthesia and regional block for thoracoscopic surgery under spontaneous respiration: a prospective cohort study. Braz J Med Biol Res. (2020) 53:e8645. 10.1590/1414-431x20198645 31859910 PMC6915876

[23] Hung WT Hung MH Wang ML Cheng YJ Hsu HH Chen JS. Nonintubated thoracoscopic surgery for lung tumor: seven years' experience with 1,025 patients. Ann Thorac Surg. (2019) 107:1607–12. 10.1016/j.athoracsur.2019.01.013 30763562

[24] Moon Y AlGhamdi ZM Jeon J Hwang W Kim Y Sung SW. Non-intubated thoracoscopic surgery: initial experience at a single center. J Thorac Dis. (2018) 10:3490–8. 10.21037/jtd.2018.05.147 30069345 PMC6051783

[25] Liu J Cui F Pompeo E Gonzalez-Rivas D Chen H Yin W. The impact of non-intubated versus intubated anaesthesia on early outcomes of video-assisted thoracoscopic anatomical resection in non-small-cell lung cancer: a propensity score matching analysis. Eur J Cardiothorac Surg. (2016) 50:920–5. 10.1093/ejcts/ezw160 27165771

[26] Al-Abdullatief M Wahood A Al-Shirawi N Arabi Y Wahba M Al-Jumah M. Awake anaesthesia for major thoracic surgical procedures: an observational study. Eur J Cardiothorac Surg. (2007) 32:346–50. 10.1016/j.ejcts.2007.04.029 17580117

[27] Hung MH Hsu HH Chan KC Chen KC Yie JC Cheng YJ. Non-intubated thoracoscopic surgery using internal intercostal nerve block, vagal block and targeted sedation. Eur J Cardiothorac Surg. (2014) 46:620–5. 10.1093/ejcts/ezu054 24585550

[28] Wang ML Galvez C Chen JS Navarro-Martinez J Bolufer S Hung MH. Non-intubated single-incision video-assisted thoracic surgery: a two-center cohort of 188 patients. J Thorac Dis. (2017) 9:2587–98. 10.21037/jtd.2017.08.96 28932566 PMC5594156

[29] Wang ML Hung MH Chen JS Hsu HH Cheng YJ. Nasal high-flow oxygen therapy improves arterial oxygenation during one-lung ventilation in non-intubated thoracoscopic surgery. Eur J Cardiothorac Surg. (2018) 53:1001–6. 10.1093/ejcts/ezx450 29253106

[30] Starke H Zinne N Leffler A Zardo P Karsten J. Developing a minimally-invasive anaesthesiological approach to non-intubated uniportal video-assisted thoracoscopic surgery in minor and major thoracic surgery. J Thorac Dis. (2020) 12:7202–17. 10.21037/jtd-20-2122 33447409 PMC7797846

[31] Chen KC Cheng YJ Hung MH Tseng YD Chen JS. Nonintubated thoracoscopic surgery using regional anesthesia and vagal block and targeted sedation. J Thorac Dis. (2014) 6:31–6. 10.1097/00003643-201406001-00238 24455173 PMC3895585

[32] Hung MH Cheng YJ Chan KC Han SC Chen KC Hsu HH. Nonintubated uniportal thoracoscopic surgery for peripheral lung nodules. Ann Thorac Surg. (2014) 98:1998–2003. 10.1016/j.athoracsur.2014.07.036 25443006

[33] Wang ML Hung MH Hsu HH Chan KC Cheng YJ Chen JS. Non-intubated thoracoscopic surgery for lung cancer in patients with impaired pulmonary function. Ann Transl Med. (2019) 7:40. 10.21037/atm.2018.11.58 30906744 PMC6389589

[34] Hung MH Hsu HH Chen KC Chan KC Cheng YJ Chen JS. Nonintubated thoracoscopic anatomical segmentectomy for lung tumors. Ann Thorac Surg. (2013) 96:1209–15. 10.1016/j.athoracsur.2013.05.065 23998413

[35] Zammit C Liddicoat H Moonsie I Makker H. Obesity and respiratory diseases. Int J Gen Med. (2010) 3:335–43. 10.2147/IJGM.S11926 21116339 PMC2990395

[36] Littleton SW. Impact of obesity on respiratory function. Respirology. (2012) 17:43–9. 10.1111/j.1440-1843.2011.02096.x 22040049

[37] Liu J Cui F Li S Chen H Shao W Liang L. Nonintubated video-assisted thoracoscopic surgery under epidural anesthesia compared with conventional anesthetic option: a randomized control study. Surg Innov. (2015) 22:123–30. 10.1177/1553350614531662 24821259

[38] Gonzalez-Rivas D Bonome C Fieira E Aymerich H Fernandez R Delgado M. Non-intubated video-assisted thoracoscopic lung resections: the future of thoracic surgery? Eur J Cardiothorac Surg. (2016) 49:721–31. 10.1093/ejcts/ezv136 25896196

[39] Wang K Sun J Gao W Chen R Wu X He Y. Feasibility, effectiveness, and safety of a novel cryo-balloon targeted lung denervation technique in an animal model. Cryobiology. (2020) 93:27–32. 10.1016/j.cryobiol.2020.03.003 32165141

[40] Gonzalez-Rivas D Fernandez R de la Torre M Rodriguez JL Fontan L Molina F. Single-port thoracoscopic lobectomy in a nonintubated patient: the least invasive procedure for major lung resection? Interact Cardiovasc Thorac Surg. (2014) 19:552–5. 10.1093/icvts/ivu209 25006214

[41] Galvez C Navarro-Martinez J Bolufer S. Reply to the editor. J Thorac Cardiovasc Surg. (2014) 148:755. 10.1016/j.jtcvs.2014.04.042 25037947

[42] Knoll H Ziegeler S Schreiber JU Buchinger H Bialas P Semyonov K. Airway injuries after one-lung ventilation: a comparison between double-lumen tube and endobronchial blocker: a randomized, prospective, controlled trial. Anesthesiology. (2006) 105:471–7. 10.1097/00000542-200609000-00009 16931978

[43] Pompeo E Mineo D Rogliani P Sabato AF Mineo TC. Feasibility and results of awake thoracoscopic resection of solitary pulmonary nodules. Ann Thorac Surg. (2004) 78:1761–8. 10.1016/j.athoracsur.2004.05.083 15511470

[44] Pompeo E Tacconi F Mineo D Mineo TC. The role of awake video-assisted thoracoscopic surgery in spontaneous pneumothorax. J Thorac Cardiovasc Surg. (2007) 133:786–90. 10.1016/j.jtcvs.2006.11.001 17320585

[45] Ambrogi MC Fanucchi O Korasidis S Davini F Gemignani R Guarracino F. Nonintubated thoracoscopic pulmonary nodule resection under spontaneous breathing anesthesia with laryngeal mask. Innovations (Phila). (2014) 9:276–80. 10.1097/imi.0000000000000075 25084248

[46] Mineo TC Sellitri F Tacconi F Ambrogi V. Quality of life and outcomes after nonintubated versus intubated video-thoracoscopic pleurodesis for malignant pleural effusion: comparison by a case-matched study. J Palliat Med. (2014) 17:761–8. 10.1089/jpm.2013.0617 24773212

[47] Gonzalez-Rivas D Fernandez R. de la Torre M, Bonome C. Uniportal video-assisted thoracoscopic left upper lobectomy under spontaneous ventilation. J Thorac Dis. (2015) 7:494–5. 25922731 10.3978/j.issn.2072-1439.2015.01.05 PMC4387442

[48] Wu CY Chen JS Lin YS Tsai TM Hung MH Chan KC. Feasibility and safety of nonintubated thoracoscopic lobectomy for geriatric lung cancer patients. Ann Thorac Surg. (2013) 95:405–11. 10.1016/j.athoracsur.2012.10.082 23245449

[49] Mineo TC Pompeo E Mineo D Tacconi F Marino M Sabato AF. Awake nonresectional lung volume reduction surgery. Ann Surg. (2006) 243:131–6. 10.1097/01.sla.0000182917.39534.2c 16371748 PMC1449981

[50] Pompeo E Rogliani P Cristino B Schillaci O Novelli G Saltini C. Awake thoracoscopic biopsy of interstitial lung disease. Ann Thorac Surg. (2013) 95:445–52. 10.1016/j.athoracsur.2012.10.043 23245450

[51] Matsumoto I Oda M Watanabe G. Awake endoscopic thymectomy via an infrasternal approach using sternal lifting. Thorac Cardiovasc Surg. (2008) 56:311–3. 10.1055/s-2008-1038632 18615384

[52] Noda M Okada Y Maeda S Sado T Sakurada A Hoshikawa Y. Is there a benefit of awake thoracoscopic surgery in patients with secondary spontaneous pneumothorax? J Thorac Cardiovasc Surg. (2012) 143:613–6. 10.1016/j.jtcvs.2011.07.067 22104684

[53] Pompeo E Rogliani P Tacconi F Dauri M Saltini C Novelli G. Randomized comparison of awake nonresectional versus nonawake resectional lung volume reduction surgery. J Thorac Cardiovasc Surg. (2012) 143:47–54, e1. 10.1016/j.jtcvs.2011.09.050 22056369

[54] Dong Q Liang L Li Y Liu J Yin W Chen H. Anesthesia with nontracheal intubation in thoracic surgery. J Thorac Dis. (2012) 4:126–30. 10.3978/j.issn.2072-1439.2012.03.10 22833817 PMC3378229

